# 360-degree pedicled inverted internal limiting membrane flap technique without face-down posturing for large idiopathic macular holes: a retrospective case series

**DOI:** 10.5935/0004-2749.2022-0252

**Published:** 2023-03-20

**Authors:** Alana Viana Alencar de Oliveira, Jefferson Souza Pinto Porto, Levy Paz Aguiar, André Luis Carvalho de Moura Bastos

**Affiliations:** 1 Department of Ophthalmology, Hospital Humberto de Castro Lima, Salvador, BA, Brazil

**Keywords:** Retinal perforations, Postoperative care, Vitrectomy, Vitreoretinal surgery, Perfurações retinianas, Cuidados pós-operatórios, Vitrectomia, Cirurgia vitreorretiniana

## Abstract

**Purpose:**

This study aimed to determine closure rates of large idiopathic macular holes treated with pars plana vitrectomy and 360-degree pedicled inverted internal limiting membrane flap without face-down posturing and define visual improvement, types of macular hole closure, and external retina integrity as secondary outcomes.

**Methods:**

This retrospective case series analyzed all patients who were treated by vitrectomy, 360-degree pedicled inverted internal limiting membrane flap, and gas tamponade, without face-down posturing postoperatively. Age, sex, time of visual acuity reduction, other ocular pathologies, and lens status were collected. The best-corrected visual acuity and optical coherence tomography results were recorded during pre- and postoperative follow-up examinations (15 days and 2 months after surgery).

**Results:**

This study enrolled 20 eyes of 19 patients, and the mean age was 66 years. Optical coherence tomography performed 2 months after surgery revealed hole closure in 19 (95%) eyes. The median best-corrected visual acuity improved from +1.08 preoperatively to +0.66 LogMAR 2 months postoperatively (p<0.001), with a median of 20 letters of visual improvement (0.4 LogMAR) on the Early Treatment Diabetic Retinopathy Study chart. V (47.36%)- and U (52.63%)-types of closure were observed.

**Conclusion:**

The 360-degree pedicled inverted internal limiting membrane flap technique, without face-down posturing, provided a high closure rate (95%), external layer recovery, and V- and U-type foveal closure contours, in addition to visual improvement in most cases of large macular holes (even macular holes >650 µm). This technique may be a viable alternative to patients in whom traditional postoperative face-down positioning for large macular hole treatment is not possible.

## Abstract (pt)

**Objetivo:**

Determinar as taxas de fechamento de buracos maculares idiopáticos grandes tratados com vitrectomia posterior e técnica de flap invertido 360 graus pediculado de membrana limitante interna, sem posicionamento de cabeça pós-operatório e definir melhora visual, tipos de fechamento do buraco macular e integridade das camadas retinianas externas como objetivo secundário.

**Métodos:**

Este estudo foi uma série retrospectiva de casos. Todos os pacientes foram submetidos a vitrectomia com flap invertido 360 graus pediculado de membrana limitante interna e tamponamento com gás, sem posição de cabeça no pós-operatório. Idade, gênero, tempo de redução da acuidade visual, outras patologias oculares e status do cristalino foram compilados. Medida de melhor acuidade visual corrigida e tomografia de coerência óptica foram registradas durante as visitas de pré e pós-operatório (15 dias e 2 meses após cirurgia).

**Resultados:**

Vinte olhos de 19 pacientes foram incluídos neste estudo. A idade média foi de sessenta e seis anos. Um total de 19 olhos (95%) atingiu fechamento do buraco, observado através das imagens de tomografia de coerência óptica após 2 meses de cirurgia. Melhor acuidade visual corrigida média aumentou +1,08 pré-operatória para +0,66 LogMAR em 2 meses de cirurgia (p<0,001), com média de 20 letras de melhora visual (0,4 LogMAR) na tabela do *Early Treatment Diabetic Retinopathy Study*. Dois tipos de fechamento do buraco foram observados: V (47,36%) e U (52,63%).

**Conclusão:**

A técnica de flap invertido 360 graus pediculado de membrana limitante interna, sem posicionamento de cabeça no pós-operatório promoveu elevada taxa de fechamento (95%), reestabelecimento das camadas retinianas externas, fechamento com contorno foveal dos tipos V e U, além de melhora visual na maioria dos casos de BMI gran des (mesmo nos buracos maiores que 650 µm). Esta técnica pode representar uma alternativa para o tratamento de buracos maculares grandes em pacientes impossibilitados de cumprir o tradicional posicionamento de cabeça pós-operatório.

## INTRODUCTION

Most macular holes (MHs) are idiopathic and affect patients in their sixth and seventh decades of life^(1)^. The beginning and progression of MHs appear to be related to tangential and anteroposterior tractions at the level of the vitreoretinal interface^(1,2)^. Since Kelly and Wendel’s introduction in 1991, pars plana vitrectomy (PPV) and posterior hyaloid removal with or without peeling of the internal limiting membrane (ILM) became the gold standard treatment for full-thickness MHs (FTMHs), with 85%-90% closure^(1,3,4)^. Globally, studies have emphasized a high and constant rate of anatomical and visual improvements in MHs with a diameter <400 µm; by contrast, only a few studies have reported MHs with a diameter >400 µm and lower closure rates or visual improvement, with closure ranging from 40% to 80%^(5,6,7,8,9,10)^. The background started to change when Michalewska et al. first reported the “inverted ILM flap technique” for the treatment of FTMHs, achieving 98% success in their 2010 study, whereas conventional vitrectomy with ILM peeling obtained only 88%, becoming a relevant macular surgery approach to reach stable visual and anatomical improvements^(11)^. With encouraging outcomes, particularly in those >400 µm, some issues related to this approach include dissociated optic nerve fiber layer (DONFL), flap stripping (14% of cases), and flap displacement during fluid-air exchange^(11,12)^. Then, Michalewska et al. reported the “temporal peeling of ILM” to reduce surgery-related damage, revealing good results just as the one introduced in 2009 for large MH repair^(13)^. Furthermore, in 2018, the Manchester Large Macular Hole Study showed an FTMH “inflection point” in standard surgery, in which MHs with a diameter ≥650 µm had a decreased success rate, suggesting that these MHs should be treated by refined approaches, such as ILM flaps^(10)^. In 2020, da Silva Tavares Neto et al. reported a case series of a pedicled technique performed for MHs <700 µm (which they denominated as very large MHs), achieving 75% (3 of 4 patients) of successfuel closure. In this technique, a large ILM peeling area (4-5 mm diameter) around the MH was realized, and the temporal ILM remained attached to the MH border^(14)^.

In most of the techniques mentioned, the face-down posturing (FDP) period (3-4 days) was advised. This positioning is recommended by most retina surgeons worldwide after MH repair^(15)^. However, the prone position places an important exclusion criterion for MH surgery in patients without physical conditions or with limiting diseases, mostly older people, because of the difficulty of maintaining the advised position. These patients could have other future perspectives considering the results of current studies in that MH surgery with and without FDP has comparable results, revealing that this postoperative advice may be reduced or even unnecessary^(15)^.

Alberti et al. conducted the first randomized controlled trial (RCT) to compare non-supine positioning (NSP) and FDP in full-thickness MH vitrectomy (ILM peeling and 15% perfluoropropane gas tamponade), revealing closure rates of 91.1% in both techniques. For MHs >400 µm, they demonstrated the non-inferiority of NSP, lack of statistical difference between NSP and FDP, and comparable visual acuity gain from both techniques, with a visual improvement of >15 Early Treatment of Diabetic Retinopathy Study (ETDRS) letters, suggesting NSP as a standard of care in most full-thickness MHs^(16)^.

In 2020, Bastos et al. conducted a study in which the nonface-down group who underwent MH surgery with SF6 tamponade had a closure rate not significantly different from that of the FDP group, i.e., 90.4% and 90.3%, respectively, suggesting that postoperative FDP was not strictly required^(1)^.

Given the need for new surgical techniques that promote similar or improved anatomical and functional success rates, with less iatrogenic intraoperative trauma and better postoperative patient comfort, this study aimed to review the surgical features of a modified (360-degree pedicled) inverted ILM flap technique, without FDP postoperatively, in patients with idiopathic MHs with a preoperative diameter of >400 µm, compare anatomical findings with previously reported approaches, and evaluate functional features in closed MHs (best-corrected visual acuity [BCVA] before and after surgery) as a secondary outcome.

## METHODS

This retrospective case series was conducted at the *Humberto Castro Lima Hospital, maintained by the Instituto Brasileiro de Oftalmologia e Prevenção da Cegueira* (IBOPC), where the anatomical success rate of surgery for large idiopathic MH correction was evaluated, between July 2018 and December 2021.

The study included patients who underwent surgery for large idiopathic MHs with the 360-degree pedicled ILM flap technique. In all cases, idiopathic MHs were detected through indirect binocular ophthalmoscopy, confirmed by spectral-domain B-scan optical coherence tomography (OCT) (Cirrus HD-OCT 4000, Carl Zeiss Meditec, Inc., CA, USA), and subjected to horizontal and vertical scans, which determined a minimum linear diameter (MLD; minimal length of the MHs parallel to retinal pigment epithelium) of >400 µm.

After the diagnostic confirmation, all patients signed a written informed consent form and underwent ophthalmologic assessments, which consisted of measuring the BCVA in the ETDRS chart (converted to logarithm of the minimum angle of resolution, LogMAR), anterior and posterior segment biomicroscopy, retinal examination with indirect ophthalmoscopy, scleral depression, applanation tonometry with Goldmann’s tonometer, and OCT, performed using a technique certified by the manufacturer.

Patients with systemic diseases that prevented surgery, diabetic retinopathy, MHs in myopic eyes, or secondary MHs (posterior to trauma, post-uveitis, and post-cystic macular edema) and patients who had undergone posterior vitrectomy were excluded from this study. Those with a follow-up <2 months were also excluded.

**Figure 1:**
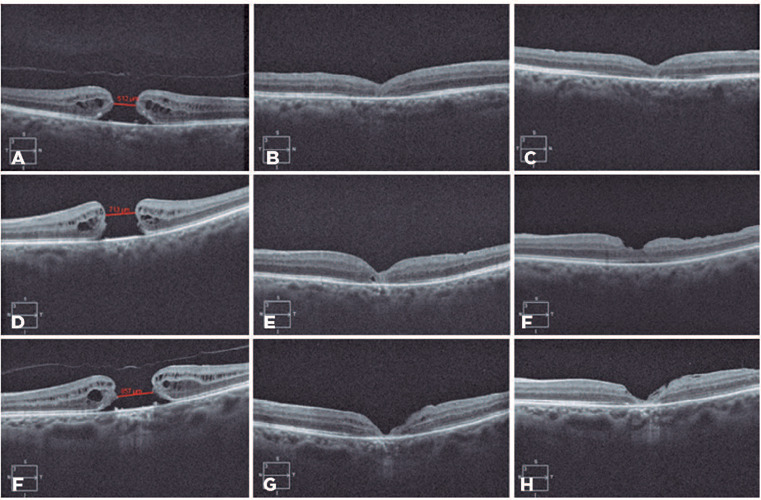
360-degree pedicled inverted ILM flap technique. (A) ILM was stained with brilliant blue solution. (B, C) The ILM was subtly peeled of inferiorly to the MH and torn circumferentially for approximately 1.5–2 disk diameter around the MH using ILM forceps. (D) The flap is left without manipulation, with 360 degrees of adhesion to the hole edges. ILM, internal limiting membrane

The entire procedure was performed by the same surgeon (ALCMB), who used a 25-gauge vitrectomy probe (Constellation Vision System, Alcon Laboratories, Inc., Fort Worth, TX, USA). After a core vitrectomy of the posterior hyaloid detachment, followed by vitreous base review to search for possible peripheral lesions, in all patients, the ILM was stained using 0.05% Brilliant Blue G (BBG, OphtBlue, R Ophtalmos, Brazil), and the ILM flap was grasped inferiorly and peeled off in a circumferential design for approximately 1.5-2 disk diameter around the MH, using Eckardt 25-gauge forceps. Then, a pedunculated flap, which was leaved 360 degrees adhered to hole edges, without further manipulation, followed by fluid-air exchange and injection of SF6 (25% mixture) gas tamponade. A diagram of the description of the surgery is illustrated in figure 1. Patients with cataracts underwent combined surgery, with phacoemulsification and foldable intraocular lens implantation (AcrySof MA60, Alcon Laboratories). Patients were instructed to avoid lying in the supine position for 7 days after the surgical procedure, without the need for FDP.

Follow-up visits were made on days 1, 15, and 60 after surgery when all patients were submitted to slit- lamp examinations and intraocular pressure measurements. The patients underwent visual acuity measurement (ETDRS chart) and spectral-domain OCT (Cirrus HD-OCT 4000, Carl Zeiss Meditec) during follow-up at days 15 and 60 postoperatively.

Anatomical surgical success was defined as MH closure on days 15 and 60 confirmed by 5-line raster B-scan OCT (horizontal and vertical scans) in each visit. During the analysis, the tomographic contour of the MH closure was evaluated and described as V-type (steep foveal contour), U-type (similar to the normal foveal contour), or flat open (foveal defect with a flattened cuff of retinal edema around borders). The recovery of the ellipzoid zone (EZ) and external limiting membrane (ELM) was also evaluated on 5-line raster B-scan OCT at the same levels during follow-up visits (horizontal and vertical central scans) through the observation of EZ and ELM defects. The evaluated layers were described as intact (complete layer restoration) or not intact (layer defect). The collected ETDRS visual acuity was converted to LogMAR. Visual success was regarded as a minimum progress of 0.3 LogMAR units (i.e., upgrade of 15 ETDRS letters).

All variables were computed and evaluated graphically for normality. Statistical analysis was performed using SPSS Statistics version 17.0 (SPSS Inc., Chicago, IL, USA). Qualitative variables were described using simple and relative frequency tables. LogMAR visual acuities before and after surgery were compared using the Wilcoxon signed-rank test. The closure type and MH size were compared using the Mann-Whitney U-rank test. A p-value <0.05 was considered statistically significant.

## RESULTS

This study enrolled 20 eyes of 19 patients. The mean patient age was 66 years. The study sample included 8 men (42.10%) and 11 women (57.89%). At least 2 months of postoperative follow-up was necessary. The duration of symptoms was not known in 7 (36.84%) patients. Among patients with a known duration of MH symptoms, the mean duration was 12.5 months. The mean MLD of the MHs was 622.6 ± 134.69 µm (408-896 µm). Moreover, 12 (60%) eyes had an MLD of <650 µm and 8 (40%) had <650 µm. Seven eyes (35%) were previously pseudophakic, and 13 (65%) were subjected to combined surgery (cataract surgery and vitrectomy). During cataract surgery in these cases, no complications such as posterior capsule rupture or descemet membrane detachment were observed.

The BCVA improved from 1.08 ± 0.36 logMAR (range 20/63-5/200 ETDRS) preoperatively to 0.78 ± 0.27 (range 20/63-10/200 ETDRS) 15 days after surgery (Z=-3.573; p<0.001), and 0.65 ± 0.26 (range 20/40-10/200 ETDRS) 2 months after surgery (Z=-3,743; p<0.001). In 7 (35%) eyes, the baseline BCVA was worse than 20/200. Fifteen days after of the surgical procedure, 10 (50%) eyes achieved BCVA of ≥20/80. Two months after surgery, 9 (45%) eyes obtained BCVA of ≥20/63. In 3 (15%) patients, vision remained at <3 LogMAR lines, and 15 (75%) patients had ≥3 LogMar lines. A total of 2 (10%) patients had worsened by 1 LogMAR line.

The anatomical success rate was 95% (19/20). MHs with preoperative MLD <650 µm (12/20) achieved 100% closure, whereas those with MLD <650 µm (8/20) achieved 87.5% closure. Two closure types of MHs, namely, U- and V-type closure, were observed in 10 (52.63%) and 9 (47.36%) eyes, respectively. No statistical significance was found in the relationship between the MH size and closure type (U=23.500; p=0.079). Regarding OCT findings among 19 eyes with closed holes, 9 (47.36%) presented with ellipsoid zone and ELM defects, and 10 (52.63%) presented only an ellipsoid zone defect. The EZ and ELM defects resolved from day 15 to day 60 of follow-up, without statistical significance (Z=-1.633; p=0.102). Complete recovery of the EZ and ELM defects was observed in 3 (15.78%) eyes 2 months after surgery. The eyes without anatomical success presented hole closure on day 15 postoperatively; however, after 2 months, the OCT revealed the total damage of the retinal layers.

No postoperative complications such as retinal detachment, epiretinal membrane, or endophthalmitis were recorded in this study.

## DISCUSSION

In the literature, large MHs have a low rate of success with traditional surgical techniques such as conventional PPV or PPV associated with ILM peeling when compared with small MHs. Williamson et al. reported a 56.3% success rate after vitrectomy for stage 4 MHs, 95.8% for stage 2 and 73% for stage 3^(17)^. Susini et al. observed a 50% success rate in MHs <500 µm^(18)^. This scenario changed after Michalewska et al. introduced the inverted ILM flap technique, which became recognized worldwide to improve the outcomes of the above situation. Through circular peeling, the ILM was not totally displaced from the retina but was left attached to MH edges^(11)^. However, this technique has some concerns, such as retinal pigment epithelium injury risk during ILM flap placement into the hole at the MH base and during OCT^(19)^. In 2015, Michalewska et al. published another version of the classic ILM flap technique, called the temporal ILM flap technique, in which the MH was covered with an ILM flap peeled only in the temporal side of the fovea, reducing surgical damage induced by peeling and maintaining a high FTMH closure rate, without a relevant difference when compared with the classic inverted peeling^(13)^.

Casini et al. compared the outcomes of two inverted ILM techniques for large FTMHs, concluding that surgical success and clinical BCVA upgrade could be reached without additional flap handling after peeling. In their study, a technique without excessive flap manipulation was introduced, in which the remaining flap that was leaved adhered only to MH borders, turning into a cone aspect, with the apex attached to the retina. Extreme care was exercised during the fluid-air exchange to prevent the opening of the cone, and air pressure was used to keep the cone lying over the hole^(20)^. Therefore, the flap could cover the MH in any ILM funnel position (temporal, nasal, upper, or lower), similar to the procedure employed in our study^(20)^. However, our postoperative care has an important difference when compared with that of Casini. In their study, they advised 3 days of FDP postoperatively, whereas our patients were instructed to avoid lying in the supine position for 7 days, without FDP, because the most important stage during surgery is to liberate the ILM tangential traction action exerted on the foveal edges, associated with the correct flap position over the hole, enabling glial Muller cells to initiate bridging at the bottom of the MH consequently^(20,21)^. Moreover, this recommendation promotes adhesion and comfort.

Within the 20 cases reported, 19 achieved anatomical closure (95%). The anatomical success rate was similar to those of some studies. Casini et al. obtained 97.6% in the classic inverted ILM flap group and 97.5% in the pedicle flap technique^(20)^. In the 2010 study of Michalewska et al., the standard technique (three-port PPV) group and inverted ILM flap group obtained closure rates of 88% and 98%, respectively^(11)^. Wang et al. reported a closure rate of 93.94% one week after surgery (tiled ILM pedicle flap transplantation); however, they included traumatic and high myopic MHs ^(22)^. To date, these studies did not evaluate the association between the minimum MH diameter and the closure rate of the MH type. Our study did not find statistically difference (U=23.500; p=0.079) when this relationship was analyzed. The high success rate in our study in MHs <650 µm (87.5%) may suggest that this technique is a good alternative in these cases.

Given that conditions that require FDP remains unclear and controversial, a meta-analysis of RCTs evaluated whether FDP is necessary for recovery from MH surgery (ILM peeling and gas tamponade). In five studies that evaluated MHs <400 µm, the subgroup meta-analysis of data showed that MH closure was significantly higher in the FDP group (odds ratio [OR] = 2.95, 95% confidence interval [CI] 1.10-7.94, p=0.03), whereas no statistically significant difference was found in studies evaluating postoperative care in MHs <400 µm (OR=1.32, 95% CI 0.39-4.49, p=0.66). They concluded that FDP may be unnecessary in MHs <400 µm, but highly recommended in those <400 µm^(23)^. In the case series by Bastos et al, no statistical difference was observed in the hole closure rates of the non-FDP and FDP groups, showing comparable anatomical and visual outcomes. In their study, the duration of MH symptoms was an anatomical and visual predictor of visual improvement (MH with duration of <12 months had 100% closure success, whereas those with <24 months had 60% closure, p=0.0012)^(1)^.

The continuity of the outer retina photoreceptor layer is an important factor for the final BCVA. Patients who had total recovery of EZ and ELM layers experienced improvement, i.e., from +0.9 to +0.6 LogMAR. Patients with EZ defect alone and both EZ and ELM defects experienced improvements, i.e., from +1.05 to +0.6 and from +1 to +0.67 LogMAR, respectively. Moreover, the ELM was the first layer to achieve anatomical morphology in most of the patients. In the study by Casini et al., 12 months after surgery, an influence of EZ defects in the mean visual acuity was observed, revealing that greater vision was achieved in patients without this defect (mean 20/22 in the Snellen chart) compared with those with EZ defect (mean 20/44 in the Snellen chart)^(20)^. Although these findings were observed in some studies, the real effect of the inverted ILM flap technique on these layers was not well established^(24)^.

In the presented technique, removal of other retina tissue, except from the ILM adjacent to the MH, is not necessary. The steps for the pedicled flap technique are quite simple, with a short learning curve, and easily reproduced. The pedicled flap is not peeled off effortlessly, which is an advantage compared with the free flap technique^(22)^. In our study, flap detachment was not observed in any step of the procedure; however, in the report by Wang et al., this complication occurred in 2 of 33 eyes^(22)^. Moreover, the position of the pedicled flap is not important during the fluid-air exchange. As an anatomical advantage of the modified inverted ILM flap technique, we can infer that a smaller ILM tissue was used to create the flap, which could leave residual ILM to eventual reoperation in the case of MH closure failure; however, further studies evaluating persistent MHs after this technique are required.

The strengths of this study included the continuous confection of the technique, which was performed by only one surgeon, regular patient visits during monitoring, and combined surgery for patients with phakic eyes; thus, cataract did not interfere in the postoperative BCVA. The retrospective data collection and short follow-up could mask the final visual acuity and anatomical results of the patients. Other limitations include the noncomparative nature of the study and the limited number of cases evaluated.

In conclusion, our study revealed that the 360-degree pedicled ILM flap technique for large idiopathic MHs provided high anatomical success rate (95% hole closure), with V- and U-types of foveal contour closure, external layer restoration, and visual improvement in most cases, representing a good alternative to the treatment of large MHs. The results emphasize that additional ILM flap handling and prone head position postoperatively may be not strictly necessary, even in MHs <650 µm, resulting in less iatrogenic damage and patient discomfort. The effect of this technique could be supplemented by prospective and randomized studies with a longer follow-up period.

## Figures and Tables

**Figure 2:**
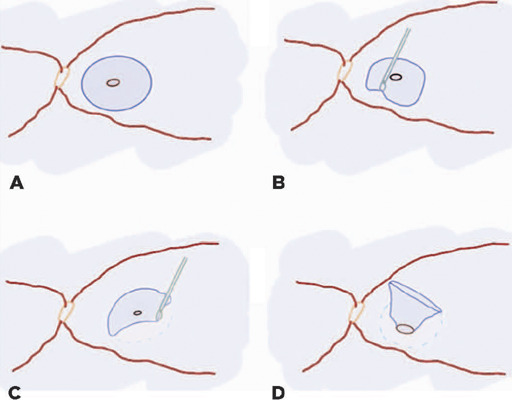
Spectral-domain OCT images of patients with full-thickness MH who were subjected to the 360-degree pedicled ILM flap (A, D, G), with 512, 713, and 857 µm respectively, and closed 15 days (B, E, and H) and 2 months (C, F, and I) after surgery, revealing recovery of the foveal architecture and discreet ellipsoid zone flaw. A visual improvement >0.3 LogMAR was obtained in these cases.

**Table 1:** Baseline and postoperative (15 days and 2 months) BCVA and external retinal layer parameters

		Baseline	15 days	2 months	p-value
Mean BCVA (LogMAR)		1.11	0.79	0.66	p<0.001
Both EZ and ELM status	Intact	–	–	3	p>0.1
	Not intact	19	19	16	
EZ status	Intact	–	–	3	
	Not intact	19	19	16	
ELM status	Intact	–	10	11	
	Not intact	19	9	8	

BCVA= best-corrected visual acuity; EZ= ellipzoid zone; ELM= external limiting membrane.

**Table 2:** Data of patients who were subjected to the 360-degree pedicled inverted ILM flap technique collected during pre- and postoperative visits

Eyes	Sex	Age	MH size (µm)	Previous BCVA	Final BCVA	MH closure
				(LogMAR)	(LogMAR)	
1	Female	69	408	1,3	1	Yes
2	Female	48	464	0.7	0.3	Yes
3	Female	69	482	0.7	0.6	Yes
4	Female	64	489	1	0.8	Yes
5	Male	67	498	1	0.3	Yes
6	Female	64	512	1	0.5	Yes
7	Female	48	536	1	0.5	Yes
8	Male	69	542	1	0.6	Yes
9	Male	65	552	1	0.5	Yes
10	Female	71	625	0.6	0.7	Yes
11	Male	71	626	1	0.5	Yes
12	Male	72	641	0.6	0.5	Yes
13	Female	59	696	1.6	0.7	Yes
14	Male	67	713	1	0.3	Yes
15	Female	56	713	1.6	0.7	Yes
16	Male	80	720	1.6	1.3	Yes
17	Male	75	736	0.5	0.6	No
18	Female	80	745	1.6	1	Yes
19	Female	61	857	1.6	0.7	Yes
20	Female	65	896	1.3	1	Yes

BCVA= best-corrected visual acuity; ILM= internal limiting membrane; MH= macular hole.
